# Anti-Graying Effects of External and Internal Treatments with Luteolin on Hair in Model Mice

**DOI:** 10.3390/antiox13121549

**Published:** 2024-12-17

**Authors:** Machiko Iida, Takumi Kagawa, Ichiro Yajima, Akihito Harusato, Akira Tazaki, Delgama A. S. M. Nishadhi, Nobuhiko Taguchi, Masashi Kato

**Affiliations:** 1 Department of Occupational and Environmental Health, Nagoya University Graduate School of Medicine, 65 Tsurumai-cho, Showa-ku, Nagoya 466-8550, Japan; machikoi@inst-hsc.jp (M.I.); kagawa.takumi.n1@s.mail.nagoya-u.ac.jp (T.K.); harusato.akihito.n8@f.mail.nagoya-u.ac.jp (A.H.); delgama.nishadhi.j7@s.mail.nagoya-u.ac.jp (D.A.S.M.N.); 2 Units of Environmental Health Sciences, Department of Biomedical Sciences, College of Life and Health Sciences, Chubu University, 1200 Matsumoto-cho, Kasugai-shi 487-8501, Japan; 3 Activities of the Institute of Innovation for Future Society, Nagoya University, Nagoya 464-8601, Japan; 4 General Research and Development Institute, Hoyu Co., Ltd., 1-12 Rouboku, Nagakute-shi 480-1136, Japan

**Keywords:** antioxidant, hair graying, model mouse, luteolin, senescence

## Abstract

Little is known about the anti-graying effects of antioxidants on hair. The anti-graying effects of three antioxidants (luteolin, hesperetin, and diosmetin) on hair were investigated according to the sequential processes of hair graying that were previously clarified in model mice [Ednrb(+/−);RET-mice]. External treatment with luteolin, but not that with hesperetin or diosmetin, alleviated hair graying in Ednrb(+/−);RET-mice. Internal treatment with luteolin also mitigated hair graying in the mice. Although both luteolin treatments had very limited effects on hair cycles, the treatments suppressed the increase in p16^ink4a^-positive cells in bulges [senescent keratinocyte stem cells (KSCs)]. Both of the treatments also suppressed decreases in the expression levels of endothelins in KSCs and their receptor (Ednrb) in melanocyte stem cells (MSCs) and alleviated hair graying in the mice. Luteolin is a special antioxidant with an anti-graying potency through improvement of age-related dysfunction in signaling between endothelins in KSCs and their receptor in MSCs. Luteolin for topical and oral use is commercially available to people in the form of supplements. Similar processes of hair graying in Ednrb(+/−);RET-mice and humans have been reported. These results are encouraging for the practical application of luteolin as a medicine with an anti-graying effect on hair in humans.

## 1. Introduction

Hair graying has a negative impression of senescence [1,2,3]. Therefore, there is a worldwide demand for medicines that can prevent hair graying or reduce the progression of hair graying [4]. However, there are limited medicines that are available for anti-graying of hair with solid scientific evidence.

A period of many years is required to investigate the effects of medicines on hair graying with aging in humans. A model animal in which age-related hair graying occurs much more rapidly than that in humans would be useful for developing medicines with an anti-graying effect on hair. Model mice [Ednrb(+/−);RET-mice] for hair graying with aging [5] have recently been established by crossing RET-transgenic mice carrying a RET oncogene [6] and heterozygously endothelin receptor-B (Ednrb)-deleted mice [Ednrb(+/−)-mice] [5]. The mechanism of hair graying was investigated with a focus on follicular keratinocyte stem cells (KSCs) [cytokeratin15 (CK15)-positive cells] and follicular melanocyte stem cells (MSCs) [dopachrome tautomerase (Dct)-positive cells] in bulges, which are stem cell pools in hair follicles in Ednrb(+/−);RET-mice [5,7,8]. The sequential hair graying processes of (1) age-related accumulation of division and self-renewing for KSCs and MSCs in every hair cycle, (2) decreased expression of endothelins (ligands) in senescent KSCs with an increased expression level of the representative senescent marker p16^ink4a^ by age-related accumulation of hair cycles, (3) decreased number of MSCs with decreased expression of endothelin receptor B (Ednrb) with aging, and (4) decreased MSCs in bulges and subsequent decreased number of descendant melanocytes in hair bulbs resulted in (5) the promotion of hair graying in the mice [5]. The major processes for hair graying, consisting of depleted survival factor of MSCs through decreased endothelins (ligands) in KSCs and decreased Ednrb (their receptor) in MSCs with aging, were shown to be comparable in model mice and humans in our previous study [5]. It might be possible to develop a medicine for alleviating hair graying if any part(s) in the entire process can be suppressed.

Luteolin (3′,4′,5,7-tetrahydroxyflavone), a plant-based flavonoid, not only has anti-aging and anti-oxidative effects [9,10,11,12] but also contributes to skin biology [13,14,15,16,17]. However, there is no direct evidence for the effects of luteolin on hair graying. In this study, the effects of luteolin and other antioxidants, including hesperetin and diosmetin, on hair graying with aging were investigated in Ednrb(+/−);RET-mice.

## 2. Materials and Methods

### 2.1. Mice

Signalings of both RET and endothelins are biological modifiers for hair growth [18,19,20] and melanin production [21]. Correspondingly, Ednrb(+/−);RET-mice [6] that were established by crossing RET-transgenic mice [22] and heterozygously endothelin receptor-B (Ednrb)-deleted mice [Ednrb(+/−)-mice] provided by the Jackson Laboratory [23] were used as model mice for hair graying [5]. Clipping [5] or shaving [18] hairs in the back skin of Ednrb(+/−);RET-mice was performed following previous studies.

### 2.2. External and Internal Treatments with Luteolin

Luteolin (Cas# 491-70-3) was purchased from LKT Laboratories (Saint Paul, MN, USA). External treatment for Ednrb(+/−);RET-mice was performed using the previously described methods [24] with a slight modification. External treatment using 200 µL of 1.0% luteolin lysed in 70% ethanol (*w*/*v*) on back skin was performed daily for 16 weeks. Internal treatment using luteolin (0.5 mg/g body weight/day) for mice was performed daily for 16 weeks according to a previously described method [25].

### 2.3. External Treatment with Hesperetin and Diosmetin

Hesperetin (Cas# 520-33-2) and diosmetin (Cas# 520-34-3) were purchased from LKT Laboratories (Saint Paul, MN, USA) [26,27]. External treatment for Ednrb(+/−);RET-mice was performed using the previously described methods [24] with a slight modification. External treatment using 200 µL of 1% hesperetin or diosmetin lysed in 70% ethanol (*w*/*v*) on the back skin was performed daily for 16 weeks.

### 2.4. Immunohistochemical Analysis

Paraffin sections were treated with 10 mM sodium citrate (pH 6.0) for antigen retrieval as previously reported. Anti-p16^ink4a/INK4A^ (1:3000, F-12, Santa Cruz Biotechnology, Dallas, TX, USA), anti-endothelins (1:100, Santa Cruz Biotechnology, Dallas, TX, USA), and anti-dopachrome tautomerase (Dct) (1:100, Santa Cruz Biotechnology, Dallas, TX, USA) were used as first antibodies. Fluorescence-labeled secondary antibodies, Alexa Fluor 594 anti-goat IgG, Alexa Fluor 594 anti-rabbit IgG, and Alexa Fluor 488 anti-mouse IgG (1:1000, Invitrogen, Waltham, MA, USA) were used for detection of target molecules. Fluorescence intensities of stained cells were evaluated using WinROOF (Mitani Corporation, Tokyo, Japan) using the method previously described.

### 2.5. Laser Capture Microdissection and Quantitative Polymerase Chain Reaction (qPCR)

Methods for laser capture microdissection [5] and qPCR [28] were previously described.

### 2.6. Statistical Analysis

Statistical differences between two groups were analyzed by the two-sided Mann–Whitney U test as previously described [29,30]. All statistical analyses were performed using JMP Pro (SAS Institute, Cary, NC, USA) [31].

## 3. Results and Discussion

**Figure 1:**
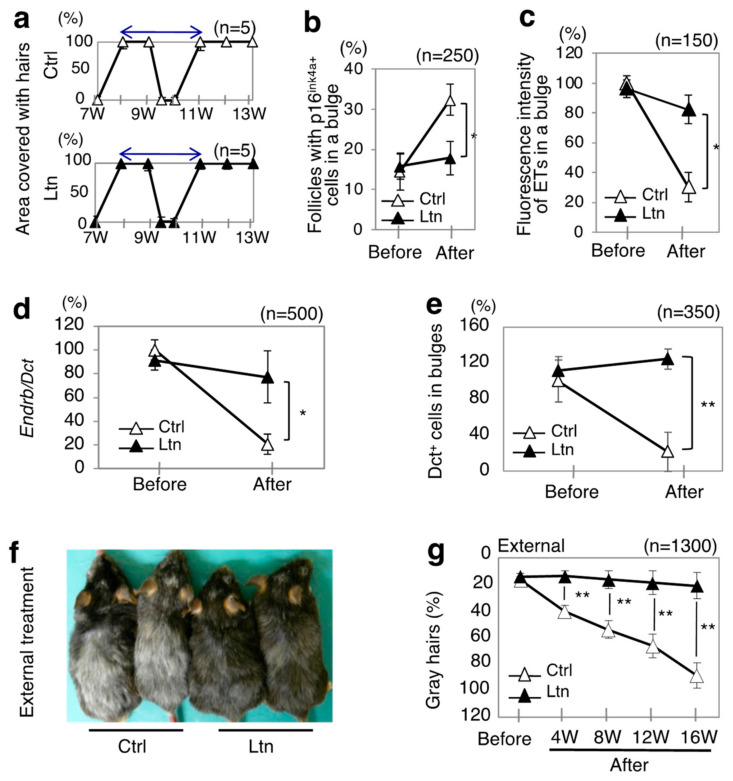
Anti-graying effect of external treatment with luteolin on hair in model mice. (**a**–**g**) Results of external treatment with luteolin (Ltn, closed triangle) (*n* = 5) or a solvent (Ctrl, open triangle) (*n* = 5) in model mice for hair graying [Ednrb(+/−);RET-mice] from 7 weeks to 13 weeks of age (**a**) and from 20 weeks of age (before treatment; Before) to 36 weeks of age (after treatment; After) (**b**–**g**) are presented. Ratios (means ± SD) of indicated items before and after treatment with 1% luteolin and after treatment with the solvent of luteolin relative to that before treatment with the solvent of luteolin in the model mice with hairs on their backs shaved once every 4 weeks are shown (**b**–**g**). (**a**) Ratios (means ± SD) of the skin area covered with hairs after clipping at 7 weeks of age (first clipping) and at 9 weeks of age (second clipping) in luteolin-treated mice (bottom) and solvent-treated mice (top). Two-way arrows show one hair cycle. (**b**) Ratios of follicles with p16^ink4a^-positive cells in telogen bulges [senescent keratinocyte stem cells (KSCs)] (*n* = 250). (**c**) Ratios of fluorescence intensity of endothelins (ETs) in telogen bulges (*n* = 150). (**d**) Ratios of Ednrb transcript expression levels to Dct transcript expression levels in telogen bulges (MSCs) isolated by laser capture microdissection (*n* = 500). (**e**) Ratios of Dct-positive cells in telogen bulges (MSCs) (*n* = 350). (**f**) Representative macroscopic appearances 16 weeks after treatment with luteolin (Ltn) or its solvent (Ctrl). (**g**) Ratios of gray hairs (*n* = 1300) for mice at indicated weeks after treatment with luteolin and the solvent. * and **, significantly different (* *p* < 0.05; ** *p* < 0.01) by the Mann–Whitney U test. W, weeks.

In this study, the anti-graying effect of luteolin on hair was investigated in Ednrb(+/−);RET-mice in which hairs on back skin were clipped (Figure 1a) or shaved (Figure 1b–g). External treatment with luteolin had no effect on hair cycles in the model mice (Figure 1a). The percentage of p16^ink4a^-positive cells in bulges in luteolin-treated mice was significantly lower than that in mice not treated with luteolin (Figure 1b). Our previous study demonstrated that the expression of other latent senescence markers, including phosphorylated p53 and p21, was undetectably low in hair follicles of wild-type mice as well as hair graying model mice at any stage or age [5]. Moreover, there were no senescent MSCs that were double positive for p16^ink4a^ and Dct in bulges, which are pools for KSCs and MSCs in follicles, in Ednrb(+/−);RET-mice [5]. Therefore, the p16^ink4a^-positive cells in bulges depicted in Figure 1b can be identified as KSCs, suggesting that luteolin suppresses the senescence of follicular KSCs.

The fluorescence intensities of endothelins in telogen bulges (Figure 1c) were significantly reduced in luteolin-treated mice compared to those in untreated mice. Similarly, the expression levels of Ednrb transcripts in Dct-positive cells in bulges (MSCs) (Figure 1d) were also decreased. Moreover, the percentages of Dct-positive cells in bulges (MSCs) (Figure 1e) and the percentages of gray hairs (Figure 1f,g) were significantly lower in the luteolin-treated group. Since endothelin signaling and Ednrb signaling act as survival factors for MSCs [5,32], these findings suggest that luteolin improved the signaling dysfunction between endothelins in KSCs and their receptor in MSCs, ultimately alleviating hair graying.

**Figure 2:**
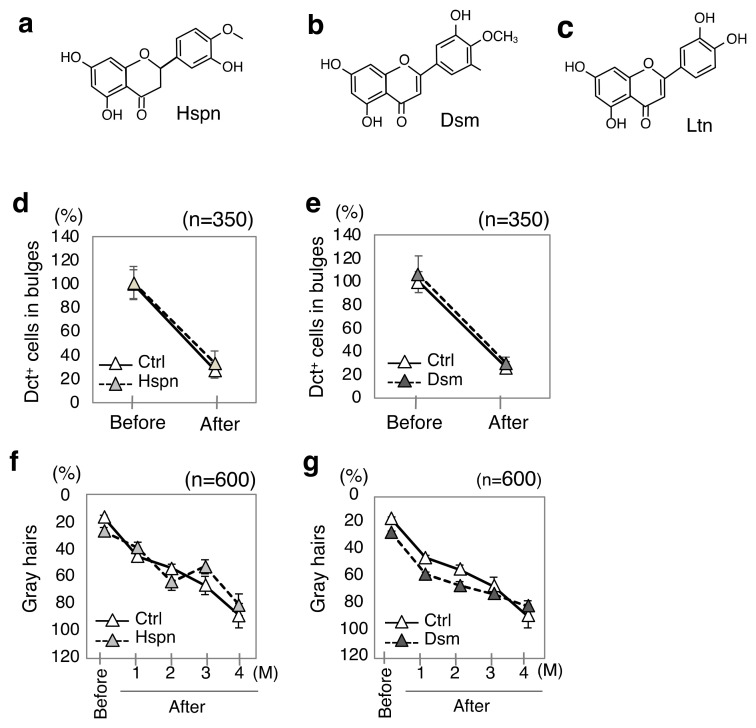
Effects of external treatment with hesperetin and diosmetin on hair graying. (**a**–**c**) Chemical structures of luteolin (Ltn), hesperetin (Hsp), and diosmetin (Dsm) are shown. (**d**–**g**) Results (means ± SD) of external treatment with 1% hesperetin (**d**,**f**) (Hspn, closed triangle, *n* = 5), 1% diosmetin (**e**,**g**) (Dsm, closed triangle, *n* = 5) and their solvent (**d**–**g**) (Ctrl, open triangle, *n* = 5), for model mice of hair graying [Ednrb(+/−);RET-mice] from 20 weeks (before treatment; Before) to 36 weeks (after treatment; After) of age are presented. Ratios of the indicated items before and after treatment with hesperetin (**d**,**f**) or diosmetin (**e**,**g**) and after treatment with their solvent relative to that before treatment with their solvent in the model mice with hairs on their backs shaved once every 4 weeks are shown (**d**–**g**). Ratios of Dct-positive cells (MSCs) in telogen bulges (**d**,**e**) (*n* = 350) and gray hairs (**f**,**g**) (*n* = 600) are presented. The Mann–Whitney U test was used because different control mice were used for treatment with different chemicals. No significant differences were found between control mice and treated mice. Note that the corresponding results regarding the effects of external treatment with luteolin on Dct+ cells in bulges (%) and gray hairs (%) are presented in Figure 1e,g.

Hesperetin and diosmetin, two plant-based flavonoids [33,34], have been demonstrated to suppress reactive oxygen species-mediated cellular damage induced by oxidants such as hydrogen peroxide and ultraviolet radiation [35,36,37,38]. Consequently, the effects of external treatments with hesperetin and diosmetin on hair graying were investigated in Ednrb(+/−);RET-mice. Despite the similar chemical structures of hesperetin, diosmetin, and luteolin (Figure 2a–c), hesperetin and diosmetin had no significant effect on Dct-positive cells (MSCs) in telogen bulges (Figure 2d,e) as well as the percentages of gray hairs (Figure 2f,g) in the model mice. These results suggest that other antioxidants (hesperetin and diosmetin) had no effect on hair graying in the mice. The differences in the preventive effects of the three chemicals on hair graying remain unclear. These variations may be influenced by the different adsorption properties of hair proteins toward the three chemicals [39]. On the other hand, the anti-graying effect of external treatment with luteolin in model mice is expected to be reproducible in humans due to the chemical similarity of hair proteins, such as keratin, between mice and humans [40,41]. Further studies focusing on proteins in hair are needed to elucidate these points.

**Figure 3:**
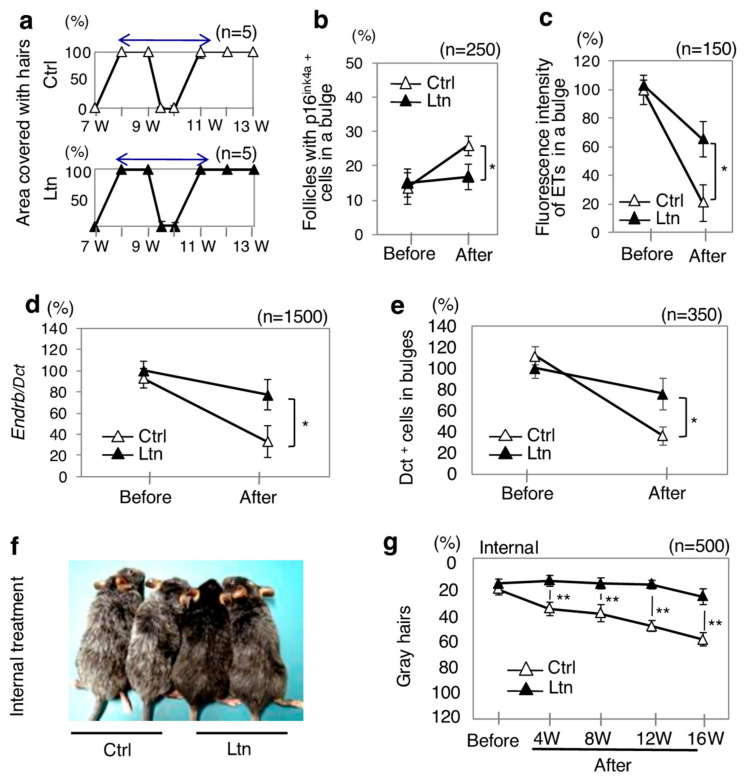
Anti-graying effect of internal treatment with luteolin on hair in model mice. (**a**–**g**) Results of internal treatment (0.5 mg/g body weight/day) with luteolin (Ltn; *n* = 5) or the solvent of luteolin (Ctrl; *n* = 5) for model mice of hair graying [Ednrb(+/−);RET-mice] from 7 weeks to 13 weeks of age (**a**) and from 20 weeks of age (before treatment; Before) to 36 weeks of age (after treatment; After) (**b**–**g**) are presented. Ratios (mean ± SD) of the indicated items before and after treatment with luteolin and after treatment with the solvent of luteolin relative to that before treatment with the solvent of luteolin in the model mice without shaving hairs (**b**–**g**) are presented. (**a**) Ratios of the skin area covered with hairs after clipping at 7 weeks of age (first clipping) and at 9 weeks of age (second clipping) in luteolin-treated mice (bottom) and solvent-treated mice (top). Two-way arrows show one hair cycle. (**b**) Ratios of follicles with p16^ink4a^-positive cells in telogen bulges [senescent keratinocyte stem cells (KSCs)] (*n* = 250). (**c**) Ratios of fluorescence intensity of endothelins (ETs) in telogen bulges (*n* = 150). (**d**) Ratios (means ± SD) of Ednrb transcript expression levels to Dct transcript expression levels in telogen bulges (MSCs) isolated by laser capture microdissection (*n* = 1500). (**e**) Ratios of Dct-positive cells in telogen bulges (MSCs) (*n* = 350). (**f**) Representative macroscopic appearances 16 weeks after internal treatment with (Ltn) or without treatment (Ctrl). (**g**) Ratios of gray hairs (*n* = 500) for mice at indicated weeks after treatment with luteolin and the solvent. * and **, significantly different (* *p* < 0.05; ** *p* < 0.01) by the Mann–Whitney U test. W, weeks.

Previous studies have demonstrated that internal treatment with luteolin exhibits antioxidative effects in both humans and mice [42,43,44,45,46]. More is known about its internal treatment than its external treatment in relation to the pharmacological effects of luteolin [47,48,49,50,51]. Therefore, it was next investigated whether internal treatment with luteolin can improve the age-related hair graying in the model mice in the condition of no hair shaving, which modulates hair cycles [5,18,19]. Internal treatment with luteolin had no effect on hair cycles (Figure 3a). However, internal treatment significantly suppressed the age-related hair graying process. This suppression was characterized by the following changes in luteolin-treated model mice compared to untreated model mice: an increase in the ratios of p16^ink4a^-positive cells in bulges (senescent KSCs) (Figure 3b), a decrease in fluorescence intensities of endothelins in telogen bulges (Figure 3c), reduced levels of Ednrb transcript expression in MSCs (Figure 3d), a decrease in the ratios of Dct-positive cells in bulges (MSCs) (Figure 3e), and a reduction in the percentages of gray hairs (Figure 3f,g). These results suggest that both external and internal treatments with luteolin potentially suppress hair graying through a similar mechanism, while internal treatment with luteolin showed a weaker anti-graying effect on hair than the effect of external treatment with luteolin.

Thus, this study indicated that external treatment with luteolin, but not that with hesperetin or diosmetin, could alleviate the hair graying in Ednrb(+/−);RET-mice, despite the fact that the chemical structures of the antioxidants are similar. This study also suggested that not only external treatment but also internal treatment with luteolin has a suppressive effect from the upstream process of decreased expression levels of endothelins in senescent KSCs to the downstream process of decreased number of follicular MSCs with decreased expression level of Ednrb, resulting in the alleviation of hair graying.

Our qPCR analysis further showed that luteolin decreased the transcript expression level of the senescent marker p16^INK4A^ (Supplemental Materials Figure S1a) and increased the transcript expression level of endothelin-1 (Supplemental Materials Figure S1b) in cultured human nontumorigenic skin keratinocytes (HaCaT cells) [52,53]. Luteolin had a limited effect on the expression levels of the endothelin-2 transcript in HaCaT keratinocytes, while the expression level of the endothelin-3 transcript was undetectably low. Our previous study demonstrated that endothelin-1, but not endothelin-2 or endothelin-3, was a key molecule involved in hair graying in Ednrb(+/−);RET-mice [1]. It is difficult to extrapolate the in vitro results derived from HaCaT keratinocytes to the in vivo findings observed in KSCs from the model mice for gray hair. However, these results suggest the potential involvement of endothelin-1 signaling in keratinocytes in mediating the effects of luteolin.

Our previous study demonstrated that the phosphorylation of histone H2AX (γH2AX), a marker of DNA damage, increased in genetically engineered model mice for hair graying [5]. Since DNA damage is often induced by oxidative stress [54,55], these results suggested a latent promotive role for oxidative stress in hair graying [56,57,58,59]. To more directly investigate this hypothesis, wild-type mice were treated with tert-butyl hydroperoxide (t-BOOH), an inducer of oxidative stress [60,61,62]. Consistent with our hypothesis, wild-type mice treated with t-BOOH developed hair graying (Supplemental Materials Figure S2a). Conversely, treatment with luteolin, an inhibitor of oxidative stress, significantly attenuated t-BOOH-induced hair graying (Supplemental Materials Figure S2a,b). These findings suggest not only the promotion of hair graying with an increase in oxidative stress but also reduction in hair graying by a decrease in oxidative stress in our model mice.

## 4. Conclusions

This study showed for the first time that both internal treatment and external treatment with luteolin prevented hair graying in Ednrb(+/−);RET-mice. This study also provided the first evidence for an anti-aging effect of luteolin on stem cells in vivo. These results suggest that luteolin is a latent medicine with an anti-graying effect on hair in model mice.

Topical and oral administration of luteolin showed an anti-graying effect on hair without exhibiting any visible signs of toxicity in mice. Similar mechanisms of hair graying in Ednrb(+/−);RET-mice and humans were shown in our previous study [5]. Therefore, this study may represent the first step toward establishing a new application of luteolin as a contributor to anti-aging and esthetic well-being in humans. To the best of our knowledge, however, no comprehensive studies have demonstrated the safety of luteolin, including its appropriate concentration, for external and internal treatments in humans. Further research is needed to investigate not only its efficacy but also its safety in human applications.

## Data Availability

The data underlying this article will be shared on reasonable request to the corresponding author.

## Supplementary Materials

The following supporting information can be downloaded at: https://www.mdpi.com/article/10.3390/antiox13121549/s1, Figure S1: Luteolin increased ET-1 expression levels with decreased expression level of p16^INK4A^ in human HaCaT keratinocytes; Figure S2: Luteolin attenuates t-BOOH-induced hair graying.
