# Supplementary material for: Anti-Graying Effects of External and Internal Treatments with Luteolin on Hair in Model Mice
Source: Antioxidants (Basel). 2024 Dec 17;13(12):1549. doi: 10.3390/antiox13121549 (PMC11673595; doi:10.3390/antiox13121549)
Supplement: Supplementary file 1 [file antioxidants-13-01549-s001.zip › antioxidants-3135360-supplementary.pdf]

Supplemental Materials

Anti-graying effects of external and internal treatments with luteolin on hair in model mice

Machiko Iida[1,3], Takumi Kagawa[1], Ichiro Yajima[1,3], Akihito Harusato[1], Akira Tazaki[1,2], Delgama A.S.M. Nishadhi[1], Nobuhiko Taguchi[3,4], Masashi Kato[1,2,3,#]

Department of [1]Occupational and Environmental Health, Nagoya University Graduate School of Medicine, 65 Tsurumai-cho, Showa-ku, Nagoya, Aichi, 466-8550, Japan. [2]Activities of the Institute of Innovation for Future Society of Nagoya University. [3]Units of Environmental Health Sciences, Department of Biomedical Sciences, College of Life and Health Sciences, Chubu University, 1200 Matsumoto-cho, Kasugai-shi, Aichi, 487-8501, Japan. [4]General Research and Development Institute, Hoyu Co., Ltd., 1-12 Rouboku, Nagakute-shi, Aichi, 480-1136, Japan.

[#]**Correspondence**: Masashi Kato M.D., Ph.D.

Department of Occupational and Environmental Health,

Nagoya University Graduate School of Medicine

Address: 65 Tsurumai-cho, Showa-ku, Nagoya, Aichi 466-8550, Japan.

Phone: +81-52-744-2122. Fax: +81-52-744-2124.

E-mail: kato.masashi.r6@f.mail.nagoya-u.ac.jp

Abbreviations

KSCs, keratinocyte stem cells

MSCs, melanocytes stem cells

CK15, cytokeratin15

Ednrb, endothelin receptor B

Supplemental Methods

Treatment with luteolin and tert-butyl hydroperoxide (t-BOOH). C57BL/6 mice (3 weeks old) were purchased from Japan SLC, Inc. The mice were treated with 250 μL of 150μM t-BOOH (tert-butyl hydroperoxide, CAS# 75-91-2; Wako Pure Chemical Co., Ltd, Osaka, Japan) to induce hair graying. Prior to t-BOOH treatment, mice received a subcutaneous injection of 300 μL of a 1% luteolin solution or a vehicle (PBS) into their clipped back skin. Four weeks later, the ratio of gray hair in the back skin was assessed by ImageJ.

Culture of HaCaT keratinocytes. Human nontumorigenic skin HaCaT keratinocytes [1] were supplied by CLS Cell Lines Service (Germany). HaCaT keratinocytes were cultured by the method previously described [2] in the presence or absence of luteolin (3 μM and 10 μM) for 3 days. The culture medium containing luteolin was renewed every day.

Supplemental Figures

Supplemental Figure S1

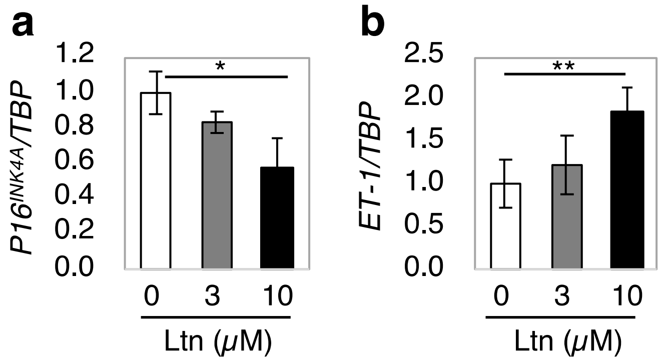

Supplemental Figure S1. Luteolin increased ET-1 expression levels with decreased expression level of p16^{INK4A} in human HaCaT keratinocytes. (a, b) Results of quantitative PCR (qPCR) for transcript expression levels of *p16^{INK4A}* (a) and *endothelin-1* (*ET-1*) (b) in human nontumorigenic skin keratinocytes (HaCaT cells) in the presence or absence of luteolin (Ltn) at 3 μM and 10 μM for 72 hours are presented. Ratios (means ± SD) were presented after the expression levels were normalized with *TATA box-binding protein (TBP)* transcript expression levels. Treatment with luteolin decreased the expression level of *p16^{INK4A}* (a) with increased *ET-1* (b) expression level. Luteolin had a limited effect on *endothelin-2* expression level, while *endothelin-3* transcript expression level was undetectably low. * and **, significantly different (*p<0.05; **p<0.01) by the Tukey-Kramer test.

Supplemental Figure S2

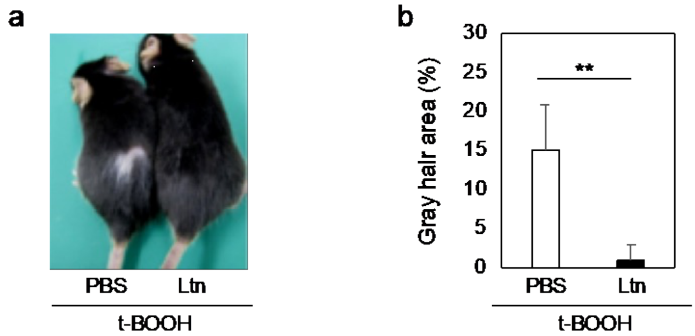

Supplemental Figure S2. Luteolin attenuates t-BOOH-induced hair graying. (a) Wild-type mice at 3 weeks of age were treated with tert-butyl hydroperoxide (t-BOOH) and luteolin (Ltn) or its solvent (PBS). An image of hair graying 4 weeks post-treatment is presented. **(b)** The ratio (mean ± SD) of the gray hair area in the back was significantly lower in mice treated with luteolin (n=6) than in PBS-treated mice (n=6) ($p<0.01$, Mann-Whitney *U* test).

Supplemental References

1. Boukamp, P.; Petrussevska, R.T.; Breitkreutz, D.; Hornung, J.; Markham, A.; Fusenig, N.E. Normal Keratinization in a Spontaneously Immortalized Aneuploid Human Keratinocyte Cell Line. *J. Cell Biol.* **1988**, *106*, 761–771, doi:10.1083/JCB.106.3.761.
2. Yajima, I.; Tazaki, A.; Ohgami, N.; Kato, M. Calcitriol Inhibits Arsenic-Promoted Tumorigenesis through Regulation of Arsenic-Uptake in a Human Keratinocyte Cell Line. *Am. J. Cancer Res.* **2022**, *12*, 5019–5028.
